# APOE ε4-related structural vulnerability in mild cognitive impairment: a subsystem-based analysis of the default mode network

**DOI:** 10.21203/rs.3.rs-9447482/v1

**Published:** 2026-05-11

**Authors:** Xiaoming Yang, Jian Lyu, Jubo Wang, Yu Quan

**Affiliations:** Second Affiliated Hospital of Xi’an Jiaotong University; Second Affiliated Hospital of Xi’an Jiaotong University; Second Affiliated Hospital of Xi’an Jiaotong University; Second Affiliated Hospital of Xi’an Jiaotong University

**Keywords:** Apolipoprotein E ε4, Mild Cognitive Impairment, Default Mode Network, Dorsomedial Prefrontal Cortex, Superior Temporal Pole, Mediation Analysis

## Abstract

**Background:**

Mild cognitive impairment (MCI) is a critical prodromal stage of progressive cognitive decline. As a core cognitive brain network, the default mode network (DMN) exhibits structural alterations in MCI that are closely associated with cognitive impairment. *Apolipoprotein E ε4 (APOE ε4)*, the strongest genetic risk factor for pathological cognitive decline, has been shown to disrupt the structural integrity of the DMN’s three core subsystems: the Midline Core, Medial Temporal Lobe (MTL), and Dorsomedial Prefrontal Cortex (DMPFC) subsystems. However, the subsystem-specific effects of *APOE ε4* and the cognitive mechanisms through which they operate in MCI remain unclear.

**Methods:**

We enrolled 176 participants from the Alzheimer’s Disease Neuroimaging Initiative (ADNI), including 116 patients with MCI and 60 cognitively normal (CN) individuals, with balanced APOE ε4 carrier status within each diagnostic group. Standardized volumes of eight DMN regions were analyzed using permutational multivariate analysis of variance (PERMANOVA) to test diagnosis-by-genotype interactions, followed by false discovery rate (FDR)-corrected univariate analyses and multiple linear regression to identify regional volume differences. Bootstrap-based mediation analysis and receiver operating characteristic (ROC) analysis were further performed to examine clinical relevance.

**Results:**

A significant diagnosis-by-*APOE ε4* interaction was observed in the global structural pattern of the DMN, primarily driven by the DMPFC and MTL subsystems. Among patients with MCI, *APOE ε4* carriers exhibited selective atrophy within the DMPFC subsystem, specifically in the gyrus rectus (REC) and superior temporal pole (TPOsup), relative to non-carriers. Critically, TPOsup atrophy mediated 30.43% of the negative effect of *APOE ε4* on global cognition, as measured by the Mini-Mental State Examination (MMSE), and was significantly associated with impaired orientation.

**Conclusion:**

The TPOsup may represent a key neural hub linking *APOE ε4* to cognitive decline in MCI and may serve as a specific imaging marker for risk stratification in this population.

## 1. Introduction

Alzheimer’s disease (AD) is a neurodegenerative disorder whose prevalence is rising steadily worldwide as populations age.(1) It is estimated that the number of affected individuals may reach approximately 153 million by 2050.(2) This trend is expected to place an enormous burden on healthcare systems, social infrastructure, and the global economy.(3) Importantly, the pathological process of AD may begin 15–20 years before the onset of overt clinical symptoms, thereby providing a critical window for early intervention.(4) Mild cognitive impairment (MCI) is generally regarded as an intermediate stage between normal aging and clinically defined dementia, particularly AD.(5, 6) Although MCI is a heterogeneous syndrome with multiple possible etiologies,(7, 8) approximately 10%–15% of individuals with MCI progress to AD each year.(9, 10) Therefore, a more comprehensive understanding of the neuropathological mechanisms underlying MCI is urgently needed to improve early detection and therapeutic intervention in AD.

At the genetic level, the *apolipoprotein E (APOE) ε4* allele is the most extensively studied and most common genetic risk factor for AD.(11, 12) Individuals carrying one copy of *APOE ε4* have approximately double the risk of developing AD, whereas those carrying two copies face a 12-fold or greater increase in risk.(13, 14) Notably, even in the presence of protective factors such as higher educational attainment, the *ε4* allele appears to diminish the effectiveness of cognitive reserve, underscoring the relative independence of its neurotoxic effects.(15, 16)

Neuroimaging studies have consistently implicated the default mode network (DMN) as a core brain network involved in the early pathological progression of AD.(17–21) Owing to its high metabolic activity at rest, the DMN may be particularly vulnerable to AD-related pathology.(22–24) Progressive disruption of the DMN’s structural and functional connectivity has been linked to cognitive decline, highlighting its importance in the preclinical and prodromal stages of AD. To further clarify the functional organization of the DMN, Andrews-Hanna et al. proposed a three-subsystem model comprising: (1) the Midline Core, which integrates information across the DMN and serves as a central hub for internally directed cognition; (2) the Medial Temporal Lobe (MTL) subsystem, which supports memory encoding and retrieval and is therefore closely related to memory impairment at the MCI stage;(25, 26) and (3) the Dorsomedial Prefrontal Cortex (DMPFC) subsystem, which is involved in affective integration, social cognition, and semantic processing, and may therefore contribute to non-memory impairments in MCI. (27, 28)

Although this model provides a valuable framework for understanding the DMN, several important knowledge gaps remain. Most neuroimaging studies of APOE ε4 have focused on traditionally defined DMN regions such as the hippocampus and posterior cingulate cortex.(29–32) First, relatively few studies have explicitly examined whether APOE ε4 exerts subsystem-specific effects within the three-subsystem DMN model, particularly within the DMPFC subsystem. Second, to our knowledge, no study has systematically investigated whether APOE ε4 produces selective neurotoxic effects on the key nodes of DMN subsystems defined by functional and anatomical connectivity, rather than on conventionally defined anatomical regions alone. Of particular importance is whether the relationship between APOE ε4 and cognitive impairment is mediated by structurally vulnerable nodes within these subsystems. These limitations hinder a more comprehensive understanding of the neural pathophysiology of cognitive decline and constrain the identification of more precise neuroimaging biomarkers.

To address these limitations, and unlike previous studies that relied on conventional region-of-interest (ROI) templates,(33, 34) we defined eight ROIs spanning the three DMN subsystems based on the functional architecture of the DMN and the pathological characteristics of AD. These regions include not only classical regions within the Midline Core and MTL subsystems, which are traditionally associated with memory-related pathology, but also less-studied regions within the DMPFC subsystem that may be involved in non-memory cognitive processes such as semantic and language-related functions. Accordingly, the present study systematically examined the differential effects of *APOE ε4* on DMN subsystems and further tested whether critical regional structural alterations mediate the association between genetic risk and cognitive performance. We hypothesized that *APOE ε4* carriers with MCI would show selective volume reductions in regions belonging to the DMPFC subsystem, and that structural alterations in these regions would partly account for the association between *APOE ε4* and cognitive decline. Overall, this study aims to delineate the subsystem-specific structural vulnerabilities associated with APOE ε4 in the prodromal stage of MCI.

## 2. Methods

The data used in this study were obtained from the Alzheimer’s Disease Neuroimaging Initiative (ADNI) database (adni.loni.usc.edu). Launched in 2003 as a public–private partnership under the leadership of Principal Investigator Dr. Michael W. Weiner, ADNI aims to determine whether serial magnetic resonance imaging (MRI), positron emission tomography (PET), other biological markers, and clinical and neuropsychological assessments can be combined to track the progression of MCI and early AD.

### 2.1. Participants

Initial screening included 205 cognitively normal (CN) individuals and 351 patients with MCI from the ADNI database.(35, 36) Participants were excluded if they met any of the following criteria: (1) poorquality MRI data (C- or D-rated based on interquartile range [IQR]); (2) carriage of the *APOE ε2* allele, to minimize its potentially protective effect against *APOE ε4*-related risk;(37, 38) or (3) a history of other major neurological disorders, psychiatric illness, or severe head trauma. After applying these criteria, 111 CN participants and 153 patients with MCI remained eligible. Diagnoses were based solely on standardized ADNI clinical criteria.(39)

**Figure 1:**
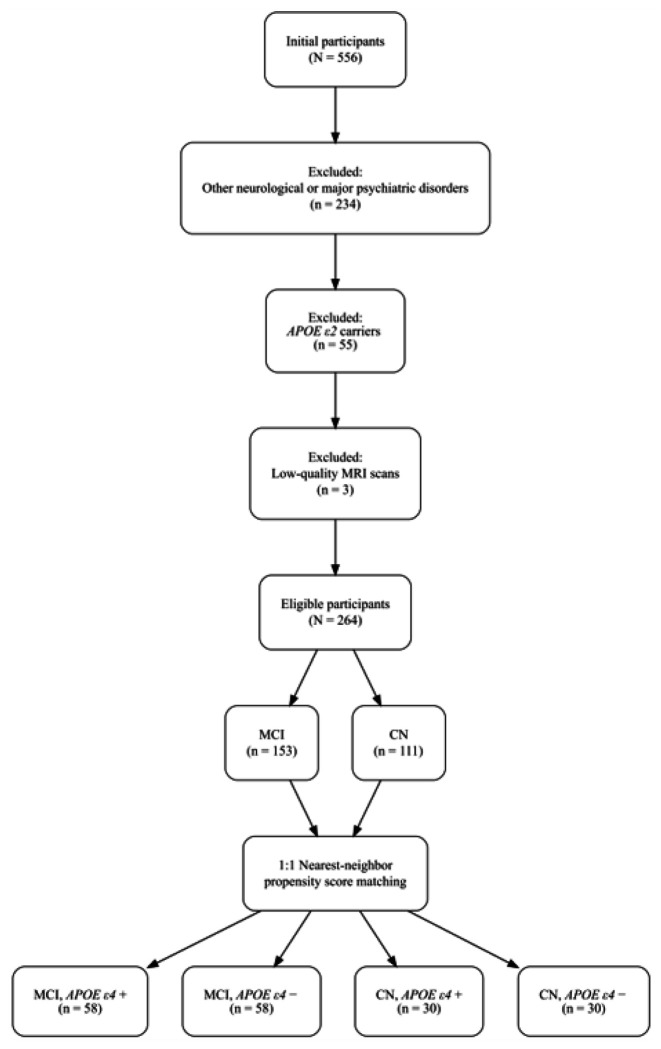
Subjects select process.

To reduce within-group imbalance, 1:1 nearest-neighbor propensity score matching (PSM) was performed separately within the CN and MCI groups. Carrier status for the *APOE ε4* allele was treated as the grouping variable, and age, sex, years of education, and body mass index (BMI) were included as covariates in the propensity score model. The caliper width was set to 0.3 times the standard deviation of the logit of the propensity score. MMSE score was not included as a matching variable because doing so could obscure the true effect of *APOE ε4* on cognition and introduce bias into subsequent mediation analyses. After matching, 58 pairs (n = 116) were obtained in the MCI group and 30 pairs (n = 60) in the CN group. Balance diagnostics confirmed that all standardized mean differences (SMDs) were below 0.1. Thus, a total of 176 participants were included in the final analyses (Figure 1).

### 2.2. MRI Data Acquisition and Processing

Baseline T1-weighted structural MRI data were downloaded from the ADNI database. All scans were acquired on 1.5T MRI scanners. Image preprocessing was performed using the Computational Anatomy Toolbox (CAT12, v12.8) implemented in MATLAB R2023a. The preprocessing pipeline was optimized for DMN subsystem analysis and included noise reduction to improve image quality, N4 bias-field correction to reduce intensity inhomogeneity caused by scanner artifacts, and segmentation of gray matter (GM), white matter, and cerebrospinal fluid. Images were spatially normalized to the Montreal Neurological Institute (MNI) 152 template (2 × 2 × 2 mm voxel size) using the DARTEL algorithm, which improves inter-subject registration by modeling fine-grained anatomical differences.(40–47)

### 2.3. Region of Interest Volume Extraction

**Figure 2:**
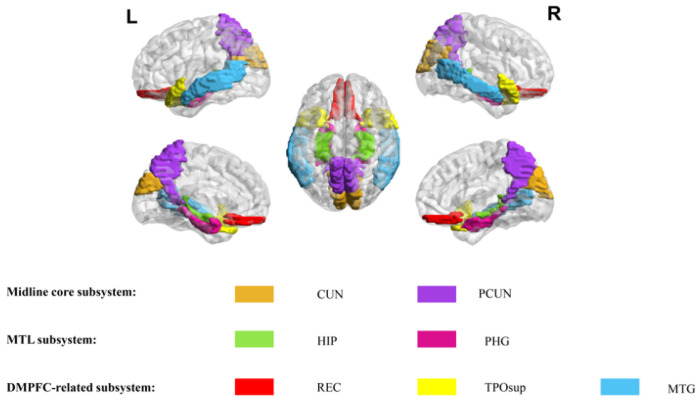
Spatial distribution of the regions of interest within the three-subsystem model of the default mode networkl.

Based on the three-subsystem DMN model proposed by Andrews-Hanna et al.(27) nd the anatomical relevance of the selected regions to the present study aims, eight ROIs were defined (Figure 2). The Midline Core subsystem included the posterior cingulate cortex (PCC) and precuneus (PCUN);(27, 48–51) the MTL subsystem included the hippocampus (HIP), parahippocampal gyrus (PHG), and frontal medial orbital cortex (FMO);(27, 52–54) and the DMPFC subsystem included the gyrus rectus (REC), superior temporal pole (TPOsup), and middle temporal gyrus (MTG).(27, 55–57) Native-space volumes of each ROI were extracted from preprocessed GM maps using the Automated Anatomical Labeling 3 (AAL3) atlas.(47) To account for inter-individual differences in head size, each regional volume was normalized to total intracranial volume (TIV) using the following formula:

NormalizingVolume=(NativeVolume/TIV)×1000

The selected ROIs are mapped onto the 3D cortical surface and grouped according to their respective subsystems. The medial temporal lobe (MTL) subsystem includes the hippocampus (HIP), parahippocampal gyrus (PHG), and frontal medial orbital (FMO). The dorsomedial prefrontal cortex (DMPFC) subsystem comprises the middle temporal gyrus (MTG), superior temporal pole (TPOsup), and gyrus rectus (REC). The Midline Core Subsystem encompasses the precuneus (PCUN) and posterior cingulate cortex (PCC). L, left hemisphere; R, right hemisphere.**]**

### 2.4. Statistical Analysis

All statistical analyses were conducted in R (version 4.2.2), with a two-tailed significance threshold of α = 0.05.

#### 2.4.1. Multivariate brain volume analysis

To evaluate the interaction between *APOE ε4* carrier status and diagnostic group, as well as their main effects on the combined normalized volumes of all eight ROIs, we first conducted a global multivariate analysis using permutational multivariate analysis of variance (PERMANOVA) implemented with the adonis2 function in the vegan package. Euclidean distance matrices and 10,000 permutations were used. Separate stratified PERMANOVA analyses were also performed for each of the three DMN subsystems. These four tests were Bonferroni-corrected, yielding a significance threshold of α = 0.0125.

#### 2.4.2. Univariate Group Comparisons

To identify which specific ROIs contributed to significant multivariate findings, univariate analyses were subsequently performed. Normality of ROI volume distributions was assessed using the Shapiro–Wilk test. For normally distributed data, independent-samples t-tests were used and effect sizes were reported as Cohen’s d; for non-normally distributed data, Mann–Whitney U tests were used and effect sizes were reported as rank-biserial correlations (r_rb). False discovery rate (FDR) correction was applied across all univariate tests, with significance defined as q < 0.05.

#### 2.4.3. Correlation and Multiple Linear Regression Analysis

Spearman’s rank correlation was used for preliminary analyses of the associations among ROI volumes, APOE ε4 status, and MMSE scores in the full sample. Multiple linear regression models were then constructed, with normalized ROI volume as the dependent variable and APOE ε4 carrier status as the primary independent variable, while adjusting for sex, handedness, TIV, and hypertension status. Hypertension status was included because a significant baseline group difference was observed (p = 0.015). Variance inflation factors (VIFs) were calculated to assess multicollinearity; all VIF values were below 5. In addition, the number of APOE ε4 alleles (0/1/2) was modeled as a continuous variable to test for a potential gene-dose effect. Finally, associations between altered regional volumes and the five MMSE subdomains—Orientation, Registration, Attention and Calculation, Delayed Recall, and Language and Visuospatial Function—were examined.

#### 2.4.4. Mediation Analysis

For variables identified as significant in the regression analyses, mediation models were constructed using the mediation package in R to test whether regional brain volume mediated the relationship between *APOE ε4* carrier status and cognitive performance. Nonparametric bootstrap resampling (1,000 iterations) was used to estimate indirect effects and their 95% confidence intervals (CIs). To examine robustness across populations and expand the dynamic range of cognitive performance, mediation analyses were performed both in the MCI subgroup and in the full matched sample. Although the Alzheimer’s Disease Assessment Scale (ADAS) score was strongly negatively correlated with MMSE (r = −0.76, p < 0.001), MMSE was selected as the primary cognitive outcome because it is more commonly used in studies of early cognitive decline.

#### 2.4.5. Exploratory ROC Analysis

Receiver operating characteristic (ROC) analysis was performed to evaluate the discriminative ability of structural, clinical, and genetic variables for differentiating MCI from CN. Three logistic regression models were constructed: a clinical model using MMSE score, a neuroimaging model using TPOsup volume, and a combined model including TPOsup volume, MMSE score, and *APOE ε4* carrier status. Age, sex, and education were included as covariates in all models. Predicted probabilities were used to generate ROC curves and calculate the area under the curve (AUC). Optimal cutoffs were determined using the maximum Youden index. DeLong’s test was used to compare AUCs across models.

## 3. Results

### 3.1. Baseline Demographic and Clinical Characteristics

After propensity score matching, the final sample comprised 176 participants, including 60 CN individuals and 116 patients with MCI. Within the CN group, 30 participants were *APOE ε4* non-carriers and 30 were carriers. Within the MCI group, 58 participants were *APOE ε4* non-carriers and 58 were carriers. Among *APOE ε4* carriers, the MCI group included 17 homozygotes and 41 heterozygotes, whereas the CN group included 2 homozygotes and 28 heterozygotes.

As shown in Table 1, no significant differences were observed between *APOE ε4* carriers and non-carriers in age, sex, education, BMI, or TIV within either diagnostic group (all p > 0.05). However, hypertension status differed significantly between *APOE ε4* subgroups in the CN group (p = 0.015). Specifically, the proportion of normotensive individuals was higher in the *APOE ε4−* subgroup (80.0%) than in the *APOE ε4+* subgroup (46.7%). Therefore, hypertension status was included as a covariate in subsequent multiple linear regression analyses.

### 3.2. APOE ε4 Effects on DMN Regional Volumes

PERMANOVA revealed a significant multivariate interaction between diagnostic group (CN vs. MCI) and *APOE ε4* carrier status on the overall structural pattern of the DMN (F = 3.24, p = 0.007), which remained significant after Bonferroni correction. Subsystem-level analyses indicated that this interaction was primarily driven by the DMPFC subsystem (F = 4.16, p = 0.003) and the MTL subsystem (F = 4.82, p = 0.002), whereas the Midline Core subsystem showed no significant interaction (F = 1.76, p = 0.163).

### 3.3. Univariate Group Comparisons.

**Figure 3:**
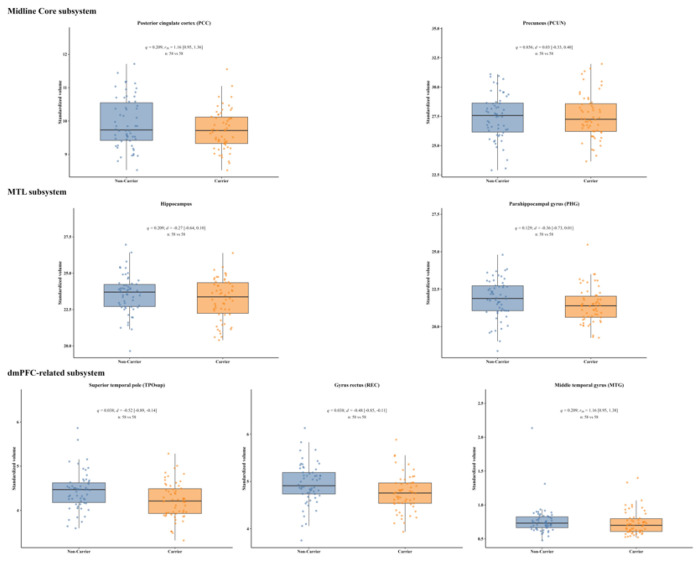
Comparison of standardized brain region volumes between *APOE ε4* carriers and non-carriers within the MCI group.

Based on the significant multivariate findings, univariate analyses were conducted to identify specific affected regions (Figure 3). In the CN group, *APOE ε4* carrier status was not associated with significant volume differences in any ROI after FDR correction (all q > 0.05).

In contrast, within the MCI group, *APOE ε4* carriers showed significant localized volume reductions in the DMPFC subsystem. Specifically, normalized volumes of the REC and TPOsup were significantly lower in *APOE ε4* carriers than in non-carriers (REC: p = 0.011, FDR q = 0.044, d = −0.48, 95% CI [−0.85, −0.11]; TPOsup: p = 0.006, FDR q = 0.044, d = −0.52, 95% CI [−0.89, −0.14]). Within the MTL subsystem, the PHG showed a trend toward lower volume in *APOE ε4* carriers (p = 0.055, d = −0.36), but this did not survive FDR correction (q = 0.148). Notably, the hippocampus—traditionally considered a key AD-related structure—did not differ significantly between groups (p = 0.153, q = 0.238). Other DMPFC regions, such as the MTG, also showed no significant differences (all q > 0.05). These findings suggest that the DMPFC subsystem may be more sensitive than the MTL subsystem to *APOE ε4*-related neurotoxicity during the early stages of cognitive impairment.

Box and scatter plots illustrate the standardized volumes of eight predefined ROIs for *APOE ε4* noncarriers (blue, n=58) and carriers (orange, n=58). The solid horizontal line inside each box represents the median, while the lower and upper hinges correspond to the first and third quartiles. Whiskers extend to the furthest data points within 1.5 × IQR. Individual subject data points are overlaid to show the distribution. Statistical significance was assessed using univariate group comparisons; q-values denote FDR-corrected p-values. Effect sizes are reported as Cohen’s *d* or rank-biserial correlation (*r_rb*), with 95% confidence intervals enclosed in brackets. Significant volume reductions in *APOE ε4* carriers are specifically observed in the gyrus rectus (REC, *q* = 0.044) and superior temporal pole (TPOsup, *q* = 0.044).Abbreviations: ROI, region of interest; MCI, Mild Cognitive Impairment; FDR, False Discovery Rate.**]**

### 3.4. Multiple Linear Regression and Sensitivity Analysis of Brain Volume and Clinical Correlations

**Figure 4:**
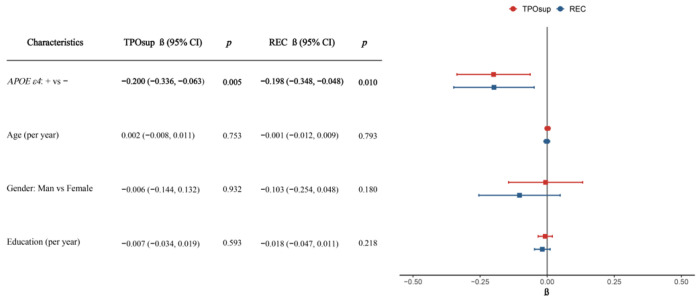
Multiple linear regression analysis of factors associated with regional brain volumes in the MCI group.

To account for potential confounding factors, multiple linear regression analyses were conducted within the MCI group. After adjusting for sex, handedness, TIV, and hypertension status (Figure 4), *APOE ε4* carrier status remained significantly associated with reduced volume in both the TPOsup (β = −0.199, 95% CI [−0.349, −0.050], p = 0.010) and REC (β = −0.185, 95% CI [−0.329, −0.042], p = 0.013). Multicollinearity diagnostics indicated that all VIF values were below 5.

In the sensitivity analysis, *APOE ε4* allele dosage was modeled as a continuous predictor. A marginal dose-dependent trend was observed for TPOsup volume reduction (Estimate = −0.109, SE = 0.055, p = 0.051). In preliminary correlation analyses across the full sample, TPOsup volume was positively correlated with global cognitive performance as measured by MMSE (Spearman r = 0.202, p = 0.007), suggesting that preserved structural integrity in this region is associated with better cognition.

The forest plot shows the unstandardized beta (β) coefficients and 95% confidence intervals (CIs) from multiple linear regression models examining the associations of *APOE ε4* carrier status (*ε4+* vs. *ε4−*), handedness (Right vs. Left), gender (Man vs. Female), hypertension status, and total intracranial volume (TIV) with the normalized volumes of the superior temporal pole (TPOsup) and gyrus rectus (REC) among participants with mild cognitive impairment (MCI) . The solid vertical line at β = 0 indicates the null effect, and the horizontal lines represent the 95% CIs. *APOE ε4* positivity was significantly and independently associated with lower TPOsup volume (p = 0.010) and lower REC volume (p = 0.013).]

### 3.5. Mediation Analysis of the Association Between APOE ε4 and Cognitive Function

#### 3.5.1. Primary Mediation Analysis in the MCI Group

**Figure 5:**
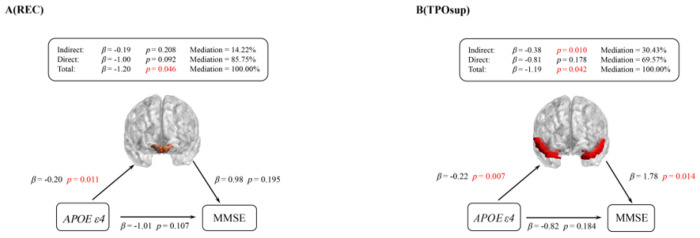
Mediation analysis of regional brain volumes on the association between *APOE ε4* status and cognitive function.

Because structural changes were predominantly observed in the MCI group, mediation analyses were first performed in this subgroup using bootstrap resampling (1,000 iterations) to test whether volumetric alterations mediated the relationship between *APOE ε4* and MMSE score (Figure 5). After adjusting for age and sex, TPOsup volume significantly mediated the association between *APOE ε4* carrier status and lower MMSE scores (indirect effect β = −0.38, 95% CI [−0.89, −0.05], p = 0.010), accounting for 30.43% of the total effect (total effect β = −1.19, p = 0.042). Although REC volume was significantly associated with genotype, it was not significantly associated with MMSE score (β = 0.98, p = 0.195) and therefore did not significantly mediate the *APOE ε4*–MMSE relationship (p = 0.208).

Path diagrams illustrate the potential mediating role of the gyrus rectus (REC, Panel A) and superior temporal pole (TPOsup, Panel B) volumes in the relationship between *APOE ε4* carrier status and MMSE scores within the MCI group. The arrows indicate the direction of the modeled effects. Unstandardized path coefficients (*β*) and corresponding p-values are presented adjacent to each path. Significant *p*-values (*p* < 0.05) are highlighted in red. The summary boxes display the unstandardized estimates for the indirect, direct, and total effects, along with the proportion of the effect mediated. (A) The REC volume does not significantly mediate the effect of *APOE ε4* on MMSE (*p* = 0.208). (B) The TPOsup volume significantly mediates the association between *APOE ε4* status and MMSE scores (indirect effect: *β* = −0.38, *p* = 0.010), accounting for 30.43% of the total effect.Abbreviations: REC, Gyrus rectus; TPOsup, Superior temporal pole; MMSE, Mini-Mental State Examination.]

#### 3.5.2. Sensitivity Mediation Verification in the Full Sample

To broaden the range of cognitive scores and test the robustness of the findings, the mediation analysis was repeated in the full matched sample. TPOsup volume remained a significant mediator (indirect effect β = −0.171, 95% CI [−0.415, −0.002], p = 0.044), accounting for 19.22% of the total effect on cognitive decline (total effect β = −0.889, p = 0.033).

### 3.6. MMSE Sub-item Regression Analysis

To identify which cognitive domain was most closely related to TPOsup atrophy, we examined associations between TPOsup volume and the five MMSE subdomain scores in the full sample. After adjusting for *APOE ε4* carrier status, multiple linear regression showed that only the Orientation subscore remained significantly positively associated with TPOsup volume after FDR correction (β = 0.676, p = 0.010, FDR q = 0.049). Although Attention and Calculation and Delayed Recall showed nominal associations with TPOsup volume, these did not survive correction for multiple comparisons (all q > 0.05, Table 2).

### 3.7. Exploratory ROC Analysis of Combined Metrics for Differentiating MCI

To evaluate the discriminative performance of structural, genetic, and clinical markers, exploratory ROC analyses were performed using logistic regression models adjusted for age, sex, and education (Figure 6). MMSE alone showed good discrimination between MCI and CN (AUC = 0.839, 95% CI: 0.780–0.898). In contrast, TPOsup volume alone showed modest performance (AUC = 0.626, 95% CI: 0.541–0.710), and APOE ε4 status alone showed poor performance (AUC = 0.541, 95% CI: 0.450–0.631). The combination of TPOsup volume and *APOE ε4* status also performed poorly (AUC = 0.621, 95% CI: 0.536–0.706) and did not significantly improve over TPOsup volume alone (DeLong’s test, p = 0.528). The full model including MMSE, TPOsup volume, and *APOE ε4* status achieved the highest AUC (0.847, 95% CI: 0.790–0.904), but this improvement over MMSE alone was not statistically significant (p = 0.362). Importantly, the full model performed significantly better than the model including only TPOsup volume and *APOE ε4* (p < 0.001), indicating that its discriminative value was primarily driven by MMSE.

The graph illustrates the diagnostic performance of three distinct predictive models: the combined model (red line; incorporating TPOsup volume, *APOE ε4* status, and MMSE), MMSE alone (green line), and TPOsup volume alone (blue line). Area under the curve (AUC) and 95% CI are provided for each classifier. Abbreviations: CN, cognitively normal; MCI, mild cognitive impairment; MMSE, Mini-Mental State Examination; TPOsup, superior temporal pole.**]**

## 4. Discussion

The present study examined whether *APOE ε4*-related structural alterations in mild cognitive impairment are evenly distributed across the default mode network or are more prominent in specific subsystems. The findings indicate that these effects are not uniform across the DMN. In this matched sample, *APOE ε4*-related volumetric differences in the MCI group were most evident in the dorsomedial prefrontal cortex subsystem, particularly in the gyrus rectus and superior temporal pole. Among these regions, TPOsup showed the most consistent association with global cognitive performance and statistically accounted for part of the relationship between *APOE ε4* status and MMSE score. Overall, these results support the view that *APOE ε4*-related structural vulnerability in MCI is better understood at the subsystem level than at the level of the DMN as a whole.

A key contribution of the study is the application of the three-subsystem DMN framework to the question of genetic risk. Previous neuroimaging studies of *APOE ε4* have primarily focused on canonical AD-related regions, including the hippocampus, posterior cingulate cortex, and precuneus, because of their established involvement in memory decline and AD pathology.(58–61) Although this literature has been highly informative, it has also tended to favor a region-centered view of vulnerability. By contrast, the present analysis was organized around functionally defined DMN subsystems. This approach showed that the interaction between diagnosis and *APOE ε4* status was detectable at the level of the overall DMN pattern and was most apparent in the DMPFC and MTL subsystems, but not in the Midline Core. This pattern does not imply that other DMN regions are uninvolved. Rather, it indicates that the structural expression of *APOE ε4* in MCI is not evenly distributed across the network and that a subsystem-based framework may capture heterogeneity that is less visible in conventional ROI analyses.(62, 63)

Within this framework, the DMPFC subsystem deserves particular attention. This subsystem has been implicated in semantic processing, self-referential cognition, affective integration, and other higher-order associative functions.(64–66) These functions rely on distributed connectivity and substantial metabolic support, both of which are relevant to *APOE ε4*-associated pathophysiology.(67) *APOE ε4* has been linked to altered lipid transport, reduced amyloid-β clearance, synaptic dysfunction, and neuroinflammatory responses.(68–70) Although the present study did not test these mechanisms directly, the regional pattern observed here is broadly consistent with the possibility that association areas within the DMN are sensitive to *APOE ε4*-related stress during the MCI stage. In this context, the DMPFC subsystem may be an informative target for further work on prodromal cognitive decline.

Among the regions examined, TPOsup emerged as the most clinically relevant in the current dataset. Its volume remained associated with *APOE ε4* carrier status after adjustment for covariates, and mediation analysis indicated that it explained a modest but nontrivial proportion of the *APOE ε4*-related difference in MMSE score. Given the cross-sectional design, this finding should be interpreted as evidence of statistical mediation rather than proof of a causal pathway. Even so, it suggests that TPOsup occupies a meaningful position between genetic status and clinical performance. This interpretation is anatomically plausible. The superior temporal pole is an association region involved in integrating semantic, contextual, and multimodal information,(71–73) and its functional profile is compatible with the idea that structural compromise in this region could contribute to broader cognitive inefficiency in MCI.

The MMSE subdomain analysis provides additional context for this interpretation. After correction for multiple comparisons, TPOsup volume was specifically associated with the Orientation subscore. Although orientation is often treated as a relatively broad screening measure, successful performance depends on the integration of temporal, spatial, and situational information rather than on episodic memory alone. This observation aligns with the known functional role and suggests that *APOE ε4*-related structural effects in MCI may extend beyond memory-dominant systems.(72–74) At the same time, this result should not be overinterpreted. The MMSE offers only a coarse assessment of cognitive domains, and the observed association would benefit from replication using more detailed neuropsychological measures.

These findings are also relevant to ongoing discussions about the relative contribution of DMPFC and MTL structures to early *APOE ε4*-related neurodegeneration. In the present study, the MTL subsystem showed a significant interaction effect at the multivariate level, but no individual MTL region, including the hippocampus, survived correction for multiple comparisons in univariate analyses. This result should not be taken as evidence against the importance of medial temporal involvement in AD. A more cautious interpretation is that, in this clinically defined and propensity-matched MCI sample, *APOE ε4*-related macroscopic volume differences were easier to detect in selected DMPFC regions than in traditional MTL structures. Several factors may contribute to this pattern. Hippocampal changes at this stage may be more readily detected by molecular, microstructural, or longitudinal measures than by cross-sectional volumetry. In addition, clinical heterogeneity within MCI may reduce sensitivity to genotype-related effects in regions that are affected by multiple pathological processes.(75–77) The subsystem-based framework may therefore complement, rather than replace, the conventional focus on the MTL.

Several limitations should be considered when interpreting the present results. First, MCI was defined using clinical criteria rather than biomarker-confirmed AD pathology. As a result, etiological heterogeneity cannot be excluded, and some participants may have had non-AD or mixed pathologies. This limitation is relevant to any structural imaging study of MCI and should temper claims about disease specificity. Second, the sample was drawn from ADNI and consisted largely of highly educated participants with limited demographic diversity, which may restrict generalizability. Third, because the study was cross-sectional, the mediation analysis should be interpreted cautiously. It identifies a statistically plausible pathway but cannot establish temporal order or causal direction. Fourth, ROI selection was theory-driven and limited to eight DMN regions. This improves interpretability but does not capture the full anatomical complexity of the DMN or its interactions with other large-scale networks. These considerations do not negate the main findings, but they define the scope within which the conclusions should be interpreted.

These limitations also point to several priorities for future research. Longitudinal studies are needed to determine whether TPOsup atrophy progresses with clinical worsening and whether it contributes to the prediction of conversion from MCI to dementia beyond standard cognitive measures. The integration of amyloid and tau biomarkers would help clarify whether the observed DMPFC-related pattern is specifically linked to AD-related pathology or reflects a broader susceptibility to cognitive decline. Replication in more diverse cohorts will also be important for testing the robustness of the findings across differences in ancestry, education, and clinical context. Finally, multimodal studies combining structural MRI with connectivity and molecular markers may help explain why TPOsup appears particularly sensitive to *APOE ε4*-related effects in this stage of impairment.

## 5. Conclusion

In summary, the present study provides evidence that *APOE ε4*-related structural variation in mild cognitive impairment is not uniformly distributed across the default mode network, but is more apparent within specific subsystems, particularly the DMPFC subsystem in the current dataset. Among the regions examined, the superior temporal pole showed the most consistent relationship with both *APOE ε4* carrier status and cognitive performance, and statistically accounted for part of the association between genetic risk and global cognition. These findings support the utility of a subsystem-based DMN framework for characterizing the neuroanatomical expression of *APOE ε4* in prodromal cognitive decline and suggest that TPOsup may be a relevant region for further investigation.

## Supplementary Material

This is a list of supplementary files associated with this preprint. Click to download.

- supplement.doc

## Figures and Tables

**Figure 6:** ROC curves for discriminating MCI from CN individuals.

**Table 1.** Demographic and clinical characteristics

	CN(N=60)				MCI(N=116)			
	
	ALL (n=60)	*APOE ε4−* (n=30)	*APOE ε4+* (n=30)	*p*	ALL (n=116)	*APOE ε4−* (n=58)	*APOE4 ε+* (n=58)	*p*
***APOE ε4* copies, n (%)**				—				—
0 copy (ε4−/−)	30 (100%)	30(100%)	0(0%)		58(100%)	58(100%)	0(0%)	
1 copy (ε4+/−)	28 (93.3%)	0 (0%)	28 (93.3%)		41 (70.7%)	0 (0%)	41 (70.7%)	
2 copies (ε4+/+)	2 (6.7%)	0 (0%)	2 (6.7%)		17 (29.3%)	0 (0%)	17 (29.3%)	
**Age, M (QI, Q3)**	77.0 (73.5, 81.0)	75 0 (74.0, 80.0)	77.5 (73.0, 81.0)	0.755	78.0 (72.0, 81.0)	78.0 (72.0, 82.0)	77.0 (73.0, 81.0)	0.980
**Gender, n (%)**				>0.999				>0.999
Female	34 (56.7%)	17 (56.7%)	17 (56.7%)		60 (51.7%)	30 (51.7%)	30 (51.7%)	
Man	26 (43.3%)	13 (43.3%)	13 (43.3%)		56 (48.3%)	28 (48.3%)	28 (48.3%)	
**Handedness, n (%)**				>0.999				0.590
Left	5 (8.3%)	2 (6.7%)	3 (10.0%)		16 (13.8%)	9 (15.5%)	7 (12.1%)	
Right	55 (91.7%)	28 (93.3%)	27 (90.0%)		100 (86.2%)	49 (84.5%)	51 (87.9%)	
**Education, M (Q1, Q3)**	16.0 (14.0, 18.0)	16.0 (14.0, 18.0)	16.0 (14.0, 18.0)	0.934	16.0 (14.0, 18.0)	16.0 (13.0, 18.0)	16.0(14.0, 18.0)	0.623
**BMI, Mean ± SD**	26.4 ±4.9	26.3 ± 5.4	26.5 ± 4.5	0.860	26.2 ±5.9	26.0 ± 5.6	26.4 ± 6.2	0.652
**TIV, Mean ± SD**	1,476.0 ± 150.0	1,489.0 ± 137.0	1,463.0 ± 164.0	0.505	1,464.0 ± 157.0	1,472.0 ± 161.0	1,456.0 ±154.0	0.588
**ADAS. M (Q1, Q3)**	8.5 (5.7, 12.2)	8.7 (5.3, 11.7)	8.2 (6.3, 13.0)	0.492	20.0 (15.0, 25.0)	19.0 (14.0, 25.0	20.0 (18.0, 25.0)	0.103
**MMSE, M (Q1,Q3)**	30.0 (28.0, 30.0)	30.0 (29.0, 30.0)	29.0(28.0, 30.0)	0.377	27.0 (24.0, 29.0)	27.5 (25.0, 29.0)	26.0 (23.0, 28.0)	**0.039**
**Hypertension Status, n (%)**				**0.015**				0.246
Normotension	38 (63.3%)	24 (80.0%)	14 (46.7%)		74 (63.8%)	34 (58.6%)	40 (69.0%)	
Stage 1 HTN	18 (30.0%)	6 (20.0%)	12 (40.0%)		36 (31.0%)	22 (37.9%)	14 (24.1%)	
Stage 2 HTN	3 (5.0%)	0 (0.0%)	3 (10.0%)		6 (5.2%)	2 (3.4%)	4 (6.9%)	
Stage 3 HTN	1 (1.7%)	0 (0.0%)	1 (3.3%)		0 (0.0%)	0 (0.0%)	0 (0.0%)	

1. *APOE ε4* carrier status was used for matching and primary analyses (carrier ≥ ε4 allele). For completeness, ε4 dosage (0/1/2 copies) is reported.

2. Normotension: Systolic Blood Pressure (SBP) < 140 mmHg / Diastolic Blood Pressure (DBP) < 90 mmHg; Stage 1 HTN: SBP 140-159 mmHg / DBP 90-99 mmHg; Stage 2 HTN: SBP 160-179 mmHg / DBP 100-109 mmHg; Stage 3 HTN: SBP ≥ 180 mmHg / DBP ≥ 110 mmHg.

2. For continuous variables: Use the t-test for normally distributed data, use the Wilcoxon test for non-normal data For categorical variables: Use Fisher’s test if the expected frequency is <5; use the χ^2^ test if ≥5.

3. CN: Cognitively Normal; MCI: Mild Cognitive Impairment, BMI: Body Mass Index, MMSE: Mini-mental State Examination; ADAS: Alzheimer’s Disease Assessment Scale; HTN: Hypertension Status; SD: Standard Deviation; M: Median; Q1: 1st Quartlie; Q3: 3st Quartile. Significant *p*-values (< 0.05) are highlighted in bold.

**Table 2.** Association between TPOsup volume and MMSE subdomains

MMSE Sub-item	β	SE	*p*	FDR *q*
Orientation	0.676	0.259	**0.010**	**0.049**
Registration	0.073	0.049	0.141	0.141
Attention & Calculation	0.369	0.186	**0.049**	0.075
Delayed Recall	0.467	0.212	**0.029**	0.073
Language & Visuospatial	0.215	0.113	0.060	0.075

Note: N = 176; *β*, unstandardized regression coefficient; SE, standard error; FDR, false discovery rate. Significant p-values (< 0.05) are highlighted in bold.

## Data Availability

The datasets generated and analyzed during the current study are available in the figshare repository under the permanent identifier 10.6084/m9.figshare.30625913.
